# Understanding condom use among unmarried female migrants: a psychological and personality factors modified information-motivation-behavioral skills model

**DOI:** 10.1186/s12889-019-6541-7

**Published:** 2019-02-21

**Authors:** Qiuming Shen, Yichen Wang, Zezhou Wang, Ying Wang, Mengyun Luo, Suping Wang, Xueqin Jiang, Yinghua Yang, Yong Cai, Meili Shang

**Affiliations:** 1 School of Public Health, School of Medicine, Shanghai Jiao Tong University, No.227, South Chongqing Road, Shanghai, 200025 People’s Republic of China; 2 Department of Hospital Infection Control, Ruijin Hospital, affiliated with the School of Medicine Shanghai Jiao Tong University, Shanghai, 200025 People’s Republic of China; 3 Shanghai Municipal Center for Disease Control & Prevention, Shanghai, 200336 People’s Republic of China; 4 Sanlin Community Health Service Center, No.375, Sanlin Road, Shanghai, 200126 People’s Republic of China

**Keywords:** Migrant, IMB model, Condom use, Woman health

## Abstract

**Background:**

In China, unmarried female migrants are vulnerable to sexual and reproductive health risks. One effective protection strategy is promoting consistent condom use (CCU).

**Methods:**

We conducted a cross-sectional study to apply the information-motivation-behavioral skills (IMB) model and modified it by addition of psychological and personal factors to examine the related factors of CCU and provide suggestions for intervention among unmarried female migrants.

**Results:**

Of all 903 eligible participants, only 13.8% of participants reported CCU in the past six months. Both the IMB model and the modified IMB model provided acceptable fit to the data. In both models, information had no direct or indirect influence on CCU (*p* > 0.05). However, behavioral skills had a positive effect on CCU (β = 0.344, *p* < 0.001 and β = 0.330, *p* < 0.001). Moreover, motivation contributed to CCU indirectly by affecting behavioral skills (β = 0.800, *p* < 0.001) and had no direct influence (*p* > 0.05). In the modified model, psychological and personality factors influenced CCU directly (β = − 0.100, *p* = 0.005).

**Conclusions:**

Our results highlight the importance of conducting CCU promotion among unmarried female migrants. Future intervention strategies should focus on both the traditional IMB model constructs and the added psychological and personality factors.

## Background

Massive migration poses severe challenges to public health worldwide. Globally, vulnerability to ill-health is a big issue towards to both international and internal migrant populations [1]. Migrants are considered to be at elevated risk for sexual and reproductive health complications and diseases, such as unintended pregnancy, induced abortions, sexually transmitted infections (STI) and human immunodeficiency virus infection/acquired immunodeficiency syndrome (HIV/AIDS) [2–4]. China’s internal migrant population (most coming from rural area to cities) has been increasing over the last decade and reached a historical record of 281 million in 2016 [5]. Separation from family, unfamiliar environments, more open attitude toward sexual behavior and limited knowledge of sexual and reproductive health enhance risky sexual behaviors among migrants [6–8]. A meta-analysis of 54 studies showed that among rural-to-urban migrants, populations floating out of or floating into provinces were observed to have a higher HIV prevalence (0.15 and 0.38%) than the general Chinese population (0.057%) [9]. Additionally, household registration (a record officially identifies a person as a resident of an area)leads to unequal access to health care, with rural migrants often lacking access to reproductive health services in cities [10]. In China, family planning services regarding reproductive health target married couples only, meaning that unmarried females are likely excluded. Previous studies have shown more than 15% of unmarried migrants have reportedly experienced unintended pregnancy [11, 12], most of which concluded in induced abortions [11–13]. Therefore, unmarried female migrants’ vulnerability to sexual and reproductive health risks requires special attention.

Condom use during sexual activity is one of the most feasible and efficient ways of preventing potential risks related to sexual and reproductive health [14]. However, consistent condom use (CCU) remains limited in migrant populations [15]. Therefore, to understand the related factors of CCU, it appears necessary to adopt a conceptually based, empirically tested, and highly generalizable model. To demonstrate what factors are related to CCU, several theories and models have been adopted, such as the Health Belief Model (HBM) [16, 17], Social Cognitive Theory (SCT) [18], Theory of Reasoned Action (TRA) [19] and Theory of Planned Behavior (TPB) [20]. Integration of these theories may yield a more complete characterization of the framework. Many of the constructs from HBM, SCT, and TRA/TPB have been incorporated into a generalized form, the Information-Motivation-Behavioral skills (IMB) model [21]. This theoretical model was developed by Fisher and it predicts AIDS-risk behavior change [22]. In particular, the IMB model explores the direct or indirect effects on behavior changes. The IMB model proposes three essential factors that bring about behavior change: information about transmission and prevention, motivation to reduce risk, and behavioral skills for performing risk reduction acts [21–23]. Information refers to knowledge concerning AIDS transmission and AIDS prevention. Motivation was measured in accord with the constructs and operation of the TRA (attitude, subjective norm, and intention). Behavioral skills includes objective skills for promoting a certain act and a sense of self efficacy for doing so. The IMB model has already been used to understand condom use in a variety of populations and its assertions have also received considerable empirical support, e.g., among male street laborers [24], students [25, 26], and sex workers [27]. However, few studies have focused on CCU by using health promotion theoretical frameworks and no study to date has applied the IMB model to investigating CCU among unmarried rural-to-urban female migrants of China.

Although the IMB model is a suitable framework for studying CCU related factors, it can be further improved. For example, the model could consider psychological problems and personality changes because they play important roles in sexual behavior among migrants, which may eventually influence their sexual and reproductive health status. Previous studies have found migrants to be more susceptible to psychological or personality problems, especially loneliness [28, 29], depression [30–32], and low self-esteem [33, 34]. These psychological and personality factors and condom use have also been found to be significantly correlated. Condom use and loneliness have been found to be negatively associated among migrant workers [29], men who have sex with men (MSM) [35] and people living with HIV [36]. Self-esteem was identified as one factor that was strongly correlated with condom use [29, 37, 38]. Moreover, depression was either correlated with condom use or a predictor of condom use in MSM [39], bisexual young men [38], and disadvantaged adults [40]. Aside from the correlation with condom use, psychological and personality factors have also been found to be related to some IMB model constructs. In particular, self-esteem was associated with condom use attitudes [41] and self-efficacy [41–43], while depression was related to attitudes toward condom use [37] and communication skills for negotiating safer sex behaviors before sexual intercourse [44]. All of these findings combined serve as an interesting supplement to the IMB model. Since psychological and personality factors cannot be integrated in information, motivation, or behavioral skill, their role in the IMB model therefore needs to be further explored. One study targeting male street laborers in urban Vietnam incorporated several new variables into a common construct labelled ‘psychosocial factors’ and assumed it worked directly or through behavioral skills to influence condom use [24].

By using structural equation modeling (SEM), we examined the associations between condom use information, motivation, and condom use behavior skills. We incorporated ‘psychological and personality factors’ as an additional construct to form a modified IMB model. Then we tested the modified IMB model to add explanatory power to the original IMB model, thus providing more evidence to guide the design of future intervention. Our hypotheses are as follows:

(1) Condom use information and motivation will indirectly affect CCU through behavioral skills. Information or motivation will also directly affect CCU. Information and motivation are interrelated.

(2) Psychological and personality factors will directly or indirectly affect CCU, and the modified IMB model is suitable for our study.

## Methods

### Participants and procedures

A cross-sectional study was performed from June to December 2015 among unmarried rural-to-urban female migrants who worked at industrial factories in Shanghai. Factories with large number of workers and high proportion of migrants were prior choices. One factory located in the city center (from Caohejing Industrial Zone) and another located in the suburban area (from Zhangjiang Industrial Zone) were selected with the assistance of local centers for disease control and prevention (CDC). Individuals were eligible if they were unmarried female migrants aged ≥18 years who had engaged in vaginal intercourse with a man over the past 6 months.

We reviewed previous literature and found that the reported CCU among participants who had reported having experienced premarital sex varied from 14.7 to 53.3% [11, 12, 45]. Assuming a CCU prevalence of 20%, α of 0.05, and a relative error of sampling of 0.15 P, we calculated that a required sample size of 938 would allow for a larger sampling error of the convenience sample method and a non-response rate of 10%.

We included female migrants who were unmarried and aged ≥18 years by following a convenience sampling process from each factory with the help of the factory managers. Participants were eligible if they answered the question on our questionnaire “Have you had vaginal intercourse with a man over the past six months?” with an answer “Yes”. We kept including participants until the number of eligible participants reached the default sample size. Altogether 2543 unmarried female migrants aged over 18 were recruited, among them 273 declined. Finally 2270 migrants participated in the research. 1028(45.3%) participants had vaginal intercourse with a man over the past six months and among them 903 (87.8%) completed the study questionnaire adequately for further analysis.

Data were collected by an anonymous and self-reported questionnaire. All participants were informed of the objectives and the procedure of the study, as well as potential risks and benefits of participating in the study. This information was written on the first page of the questionnaire, which the participants signed if they agreed to be enrolled in the study. Each questionnaire was completed in a private room. There was one investigator in each room, who would answer any questions if the participants had when finishing the questionnaire. The questionnaire was completed in approximately 30 min and respondents were given 30 RMB (approximately 4.5 USD) for their participation after completion.

## Measures

### Socio-demographic variables and reproductive health status

Socio-demographic information were collected, including age, education, income level, and length of employment in Shanghai. Reproductive health status included experience of unintended pregnancy, induced abortion, and STI.

## IMB constructs

### Information

Condom use information was measured for two items with response options of “yes”, “no”, or “do not know” from the HIV-KQ-18 scale [46]. We only adopted the items about condom use from the whole HIV Knowledge Questionnaire (18 items) because information are likely to have a association with behavior when they both are measured at the same level of specificity. Information was scored according to the number of correct responses. Items to which the participants did not know the answer were scored as incorrect; e.g., “There is a female condom that can help decrease a woman’s chance of getting HIV.” and “Using Vaseline or baby oil with condoms lowers the chance of getting HIV.” Individual question scores were summed and converted into a total score labelled as Information. (Cronbach’s alpha coefficient = 0.510; range 0–2). Higher scores indicated greater knowledge about condom use.

### Motivation

We adopted three indexes: attitudes toward perception of condom use, intentions to act, and social norm, to measure condom use motivation. These three indexes were constructed from answers rated according to a 5-point Likert scale. The first index regarding attitude toward condom use contains three items (“I think condoms are an excellent means of contraception”, “Condom use ruin the sex act” and “I feel embarrassed when suggesting using a condom”). After negatively-worded items were reverse-scored, the sum of the three items’ scores was converted into a total score labelled as Attitude, where 1 = completely disagree, and 5 = completely agree. Higher scores indicated greater positive attitude toward condom use (Cronbach’s alpha coefficient = 0.696; range 3–15). The second index of intentions to act contains four items (e.g., “the likelihood of using as condom during sex,” and “the likelihood of talking about condom use with partners”). The sum of the four items’ scores was converted into a total score labelled as Intention, where 1 = very unlikely, and 5 = very likely. Higher scores indicated that one is more likely to use a condom (Cronbach’s alpha coefficient = 0.727; range 4–20). The third index was social norm, which contains two items (i.e., “What do people that you respect think about using condoms every time if you have sex” and “What do your partner think about using condoms every time when you have sex with him”). The sum of the two items’ scores was converted into a total score labelled as Social Norm, where 1 = very supportive, and 5 = not supportive. Higher scores indicated higher social norms toward condom use (Cronbach’s alpha coefficient = 0.538; range 2–10).

### Behavioral skills

Behavioral skill was measured by two indexes constructed from answers rated according to a 5-point Likert scale. The first index was objective skills for promoting condom use, comprising four items [47], e.g., How often did you “make it clear that I would not have sex if condoms are not used.” and “tell partner that we both would be safer from disease if we used a condom”. The sum of the four items’ scores was converted into a total score labelled as Skills, where 1 = never, and 5 = always. Higher scores indicated higher levels of behavioral skills for condom use (Cronbach’s alpha coefficient = 0.830; range 4–20). The second index was a sense of self efficacy for using a condom, which contains two items (i.e., “I am confident that I can talk about condoms with my partner,” and “I am confident that I can convince my partner to use a condom even if he doesn’t want to”). The sum of the two items’ scores was converted into a total score labelled as Self Efficacy, where 1 = not at all confident, and 5 = extremely confident. Higher scores indicated the participant was more confident to use a condom (Cronbach’s alpha coefficient = 0.665; range 2–10).

## Psychological and personality factors

### Depression

Depressive symptoms were measured using 20 items from the Center for Epidemiologic Studies Depression Scale [48], according to a Likert-type scale comprising 16 positive-scoring and 4 reverse-scoring items. Participants were asked how often they had experienced depressive symptoms within the past week, where 0 = rarely or none of the time/less than 1 day, and 3 = most or all of the time/5–7 days. Higher summed scores indicated higher depression severity (Cronbach’s alpha = 0.877; range 0–60).

### Self-esteem

Self-esteem was measured using 10 items from the Rosenberg Self-Esteem Scale [49, 50], with responses rated according to a Likert-type scale. Five items were positively-worded, while the remaining five were negatively-worded. Participants were asked to rate the extent to which they agreed or disagreed with each item, where 0 = strongly disagree, and 3 = strongly agree. Positively-worded responses were reverse-scored so that all item response scores were summed, with higher total scores indicating lower self-esteem (Cronbach’s alpha = 0.773; range 0–30).

### Loneliness

Loneliness was measured using six positive-scoring and two reverse-scoring items from a simplified version of the UCLA Loneliness Scale [51], a Likert-type scale. Participants were asked to rate how often they had experienced the feeling of loneliness, with 1 = never, and 4 = most of the time. A higher summed score indicated a higher level of loneliness (Cronbach’s alpha = 0.688; range 8–32).

### Condom use behavior

Consistent condom use is defined as using a condom during every sexual intercourse encounter within the past 6 months prior to our study. Consistent condom use was measured by the question, “In the past 6 months, how often did you use condoms when you had sex with any partner?” Responses were rated according to a 5-point scale, where 1 = never, and 5 = every time. A higher score indicated a higher frequency of condom use.

#### Statistical analysis

SPSS 22.0 (IBM Corp. Released 2013. IBM SPSS Statistics for Windows, Version 22.0. Armonk, NY: IBM Corp) was used for data cleaning, coding, and preliminary analysis. Descriptive statistics included mean values and standard deviations (SD) for continuous variables and percentages for binary and categorical variables. To evaluate the correlations between the observed variables, Pearson correlation was used. The initial and modified IMB models were both examined by the SEM using the Amos 23.0 (Arbuckle, J. L. (2014). Amos (Version 23.0) [Computer Program]. Chicago: IBM SPSS). The normality test (SKEW < 3) justified the use of the maximum likelihood estimation in our analysis [52]. The model’s fit was examined by using the maximum likelihood chi-square values/degrees of freedom ratio (*χ*^2^/df), the comparative fit index (CFI) and the root mean square error of approximation (RMSEA) [53]. A CFI value > 0.9 and a RMSEA value < 0.08 indicated an acceptable fit of the model. A non-significant model chi-square test or a *χ*^2^/df ratio of ≤5 suggested an acceptable model fit [54]. Mediation effects were tested using bias-corrected bootstrap method (bootstrap BC) by Amos, which is advantageous in identifying both direct and indirect effects. The resample process is repeated for a total of 1000 times [55].

## Results

### Participant characteristics

The socio-demographic characteristics and sexual and reproductive status of the participants are described in Table 1. A total of 903 unmarried rural-to-urban female migrants completed all measures in the questionnaire. The participants’ mean age was 23.4 years old (SD = 2.7; range 18–36). For most participants, the highest education level attained was senior high school (*N* = 627, 69.4%) and 61.8% had worked in Shanghai for more than 1 year.

**Table 1:** Participants’ social-demographic, and sexual and reproductive health characteristics (*N* = 903)

Characteristic variables	Number	Percent(%)
*Age(Years)*		
18–20*	69	7.6
> 20	834	92.4
*Education*		
Junior high school and below	267	29.6
Senior high school	627	69.4
College and above	9	1.0
*Type of hometown*		
City	256	28.3
Rural	647	71.7
Average monthly income level(CRMB**)		
< 3200	280	31.0
3200–4800	585	64.8
> 4800	38	4.2
*Length of employment in Shanghai (Year)*		
≤ 1	345	38.2
>1	558	61.8
*Experience of unintended pregnancy*		
Yes	270	29.9
No	632	70.1
*Experience of induced abortion*		
Yes	255	28.2
No	648	71.8
*Experience of STI*		
Yes	6	0.7
No	897	99.3
*Condom use frequency in past six months*		
Never	88	9.7
Seldom	127	14.1
Sometimes	269	29.8
Most of the time	294	32.6
Every time	125	13.8

*In China, 20 years old is the youngest legal age for marriage among women. Doi:10.1371/journal.pone.0062787.t001

**CRMB: Chinese Yuan; 6.67 CNY = 1 USD

In total, 270 participants reported having had experienced unintended pregnancy (29.9%), among whom 94.4% reported having had experienced induced abortion. Only 13.8% of participants reported CCU in the past six months.

### Correlations among study variables

Scores on model variables and their bivariate correlations are summarized in Table 2. The majority of these variables were significantly related to one another. Most of the variables in the initial IMB model, except for Social Norm (r = 0.021, *p* > 0.05), were positively correlated with CCU. Skills and Self-efficacy of behavioral skill were the most highly correlated variables with CCU among all model variables (r = 0.387, *p* < 0.001; r = 0.315, *p* < 0.001). All additional psychological and personality variables were negatively correlated with CCU. The absolute value of the correlation coefficient ranged from 0.067 to 0.765.

**Table 2:** Descriptive statistics and correlations among model variables

	mean	SD	I	II	III	IV	V	VI	VII	VII	IX	X
I Information (Range: 0–2)	1.26	0.03	1									
II Attitude (Range: 3–15)	10.67	2.28	0.298^c^	1								
III Intention (Range: 4–20)	15.72	2.60	0.190^c^	0.286^c^	1							
IV SocialNorm (Range: 2–10)	8.39	1.23	−0.041	0.130^c^	0.120^c^	1						
V Skills (Range: 4–20)	9.83	3.73	0.211^c^	0.483^c^	0.274^c^	0.111^b^	1					
VI SelfEfficacy (Range: 2–10)	5.08	1.92	0.229^c^	0.479^c^	0.295^c^	0.131^c^	0.765^c^	1				
VII SelfEsteem(reversed) (Range: 0–30)	11.44	3.93	−0.027	−0.096^b^	0.001	0.029	−0.132^c^	− 0.117^c^	1			
VII Depression (Range: 0–48)	13.75	9.31	−0.007	0.022	0.065	0.084^a^	0.026	0.016	0.500^c^			
IX Loneliness (Range: 8–32)	15.04	3.93	−0.076^a^	−0.118^c^	0.078^a^	0.028	−0.067^a^	−0.039	0.502^c^	0.585^c^	1	
X CCU (Range: 1–5)	3.27	1.16	0.163^c^	0.265^c^	0.119^c^	0.021	0.387^c^	0.315^c^	−0.098^b^	−0.078^a^	−0.105^b^	1

SD: Standard deviation

a: *p* < 0.05; b: *p* < 0.01; c: *p* < 0.001

### Initial model testing

**Fig. 1:**
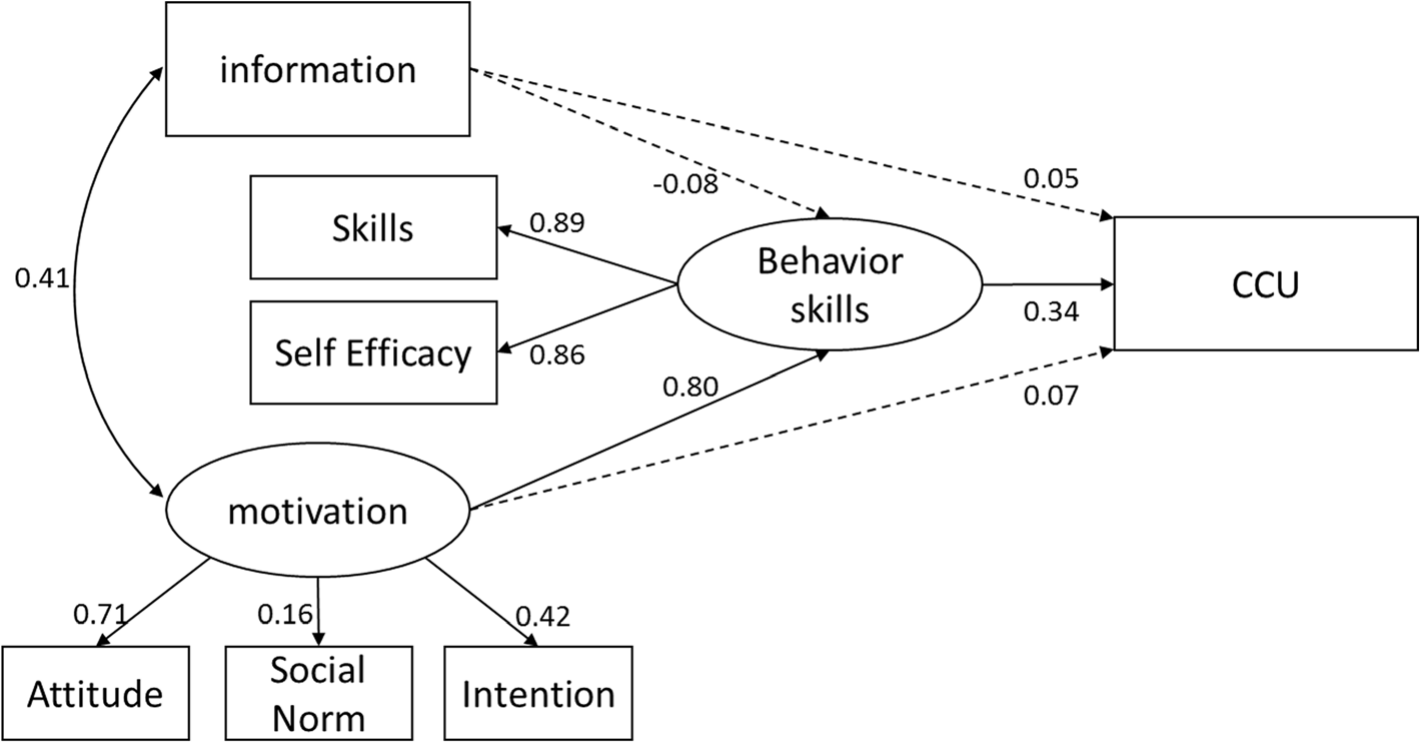
The initial information–motivation–behavioral skills model of CCU. Circles represent latent variables and rectangles represent single-item indicators. Single arrows represent standardized regression coefficients and multi-headed arrows represent standardized correlations. Solid-lined curves represent statistical significance. Dotted lines represent a lack of statistical significance

The initial model outcomes with estimates for regression weights, correlations, and path coefficients are presented in Fig. 1. Despite a significant *χ*^2^ of 33.078 (*p* < 0.001), which was subject to sample size, the *χ*^2^/df ratio, the value of CFI, and the RMSEA was 3.308, 0.984, and 0.051, respectively; these data combined indicated an acceptable model fit. The full model predicted 17.1% of the variance for CCU.

As expected, behavioral skill directly influenced CCU (β = 0.344, *p* < 0.001). We also found that motivation (β = 0.800, *p* < 0.001) was predictive of behavioral skills, which in turn predicted CCU. However, information and motivation did not directly influence CCU (β = 0.050, *p* > 0.05; β = 0.069, p > 0.05). Furthermore, paths from information to behavioral skills were not observed to be significant. Finally, information and motivation were found to be correlated (r = 0.410, *p* < 0.001).

### Modified model testing

**Fig. 2:**
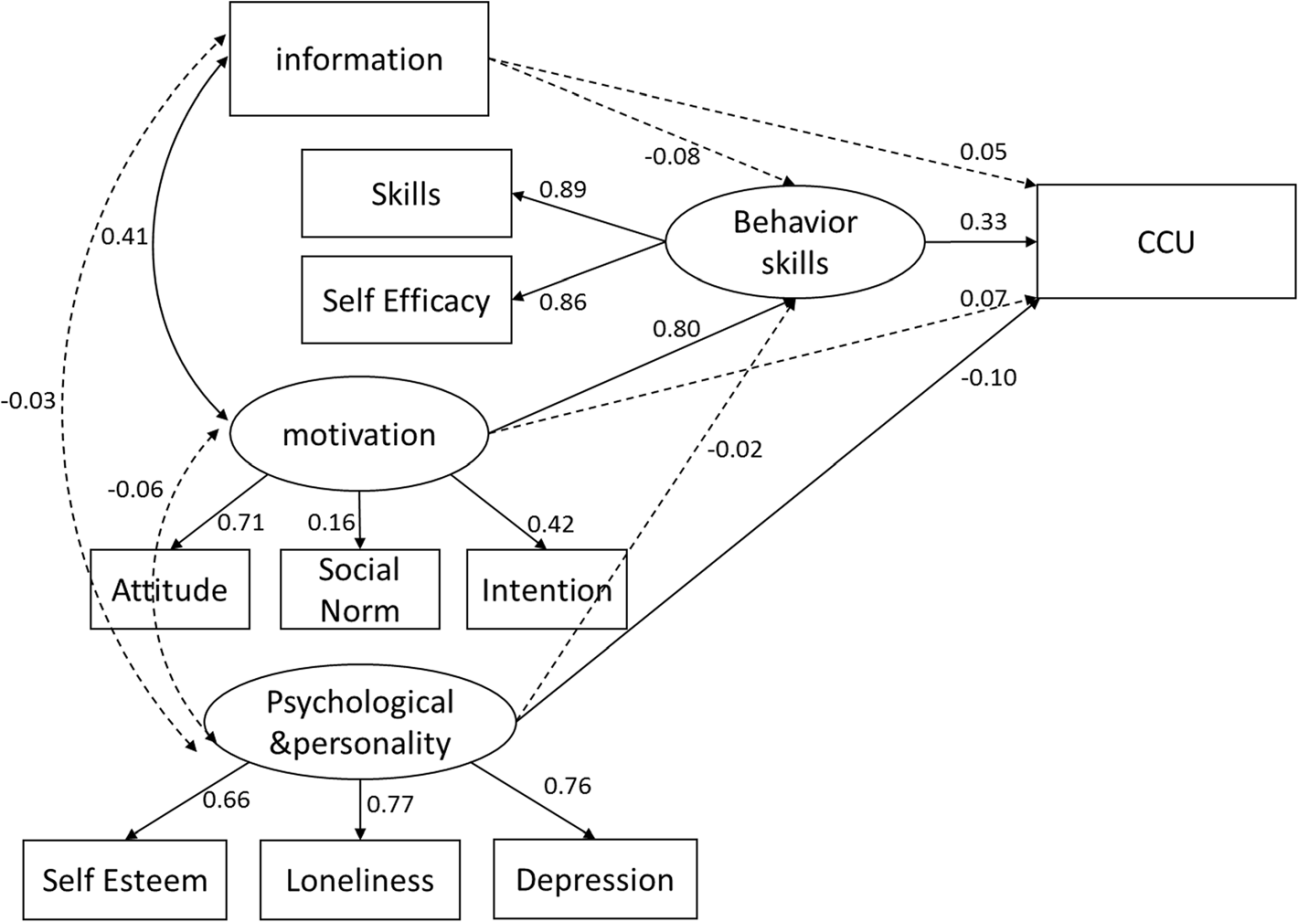
The modified information–motivation–behavioral skills model of CCU. Circles represent latent variables and rectangles represent single-item indicators. Single arrows represent standardized regression coefficients and multi-headed arrows represent standardized correlations. Solid-lined curves represent statistical significance. Dotted lines represent a lack of statistical significance

The outcomes of the modified model on psychological and personality factors are presented in Fig. 2. Despite a significant *χ*^2^of 110.592 (*p* < 0.001), which was subject to sample size, the *χ*^2^/df ratio, the value of CFI, and the RMSEA was 4.096, 0.963, and 0.059, respectively; all these data combined indicated an acceptable model fit. The modified model predicted 18.1% of the variance for CCU.

According to our results, behavioral skills (β = 0.330, *p* < 0.001) and psychological and personality factors (β = − 0.100, *p* = 0.005) directly influenced CCU. Moreover, motivation (β = 0.800, *p* < 0.001) was predictive of behavior skills, which in turn predicted CCU. Information and motivation were also observed to be correlated (r = 0.411, *p* < 0.001).

### Testing of mediation effect

The mediation test results for the modified IMB model are summarized in Table 3. The indirect effect of motivation mediated through behavioral skill on the CCU in our modified IMB model was 0.287 (*p* < 0.01). The direct effect of psychological and personality factors and CCU was − 0.016 (*p* < 0.01). No direct effect between motivation and CCU nor an indirect effect between psychological and personality factors and CCU were evident.

**Table 3:** Effects of information, motivation, and psychological and personality factors mediated through behavioral skill on CCU examined according to bootstrap BC

	X on BS	BS on CCU	indirect effect X on CCU	direct effect X on CCU	total effect X on CCU
information	−0.356	0.116**	−0.041	0.073	−0.031
motivation	2.471**		0.287**	0.074	0.361**
psychological and personality factors	−0.012		− 0.001	−0.016**	− 0.017**

*: *p* < 0.05; **: *p* < 0.01

## Discussion

Psychological and personality factors have rarely been considered when a theory-based framework is applied to understand condom use. In this study, we examined whether or not the IMB model and the modified IMB model can be used to predict CCU and how these models’ constructs influence CCU among unmarried rural-to-urban female migrants in Shanghai. Our research showed that both the IMB model and the modified IMB model appeared to be suitable for predicting CCU in the studied population. Further, motivation, behavioral skill, and psychological and personality factors all influenced CCU.

Our findings confirm that unmarried rural-to-urban female migrants are a high-risk group in terms of sexual and reproductive health. The rates of unintended pregnancy and induced abortion among unmarried female migrants were 29.9 and 28.2%, respectively. These rates are higher than that of previous regional findings, including 15.3 and 14.6% in Shanghai in 2012 [12], 10.4 and 9.8% in Qingdao in 2012 [56], and 18.4 and 17.7% in Shanghai in 2013 [11], respectively. Unprotected sex and failure of contraceptive methods are considered the main reasons for unintended pregnancies. In our study, the percentage of CCU among unmarried rural-to-urban female migrants in Shanghai during the 6 months prior to the survey was 13.8%, which was lower than that in two studies conducted in Shanghai (14.7% [11], 53.3% [12]) and another in Guangxi (26.4% [45]). The difference may be due to two reasons. One is that our study measured CCU within the last 6 months, which was a much longer period than those of previous research. Another reason is that the actual prevalence of CCU in our participants was lower than that of previous studies, which reflects the higher rate of unintended pregnancy and induced abortion in our study.

In both models examined in our study, information was not associated with behavioral skills or CCU. This inconsistency between information and behavioral skills [57, 58] and between information and HIV-preventative behavior [27, 59] has also been observed in many other studies. According to Fisher, information is both necessary and sufficient for relatively uncomplicated behavior changes (e.g., avoiding sexual contact). However, although information is necessary, it is not a sufficient condition for a complicated behavior change (e.g., CCU) because many other factors may play more important roles [22, 60]. Besides, information may impact initial behavioral change rather than maintain such behavior over time, which indicates information’s impact on maintaining condom use may be weak [61]. In addition, condom use (and condom skills) involves communication and negotiation with a partner, but previous studies have suggested that one partner’s information may have a low impact on dyadic behavioral change [62]. Additional evidence has shown that due to power dynamics between men and women, female migrants usually take a subordinate position in sexual interactions, which further lowers the impact on behavioral change [63–65].

Consistent with our hypothesis, in both models, behavioral skills directly influenced CCU, while motivation contributed to CCU indirectly by affecting behavioral skills. However, no direct influence between motivation and CCU was found. Unmarried rural-to-urban female migrants who possess higher levels of behavior skill were observed to more likely perform CCU, while highly motivated individuals were more likely to acquire the requisite skills to perform CCU. These results highlight the crucial role of behavioral skills. Interventions targeted at enhancing self-efficacy and at promoting condom use skills may have a strong effect on CCU. However, to the best of our knowledge, no published study has focused on intervention for both CCU self-efficacy and condom use skills among unmarried rural-to-urban female migrants, which should be addressed in future studies.

In the modified model, while psychological and personality factors influenced CCU directly, they were not mediated by behavioral skills that affected CCU. Participants with higher levels of depression, higher levels of loneliness, and lower levels of self-esteem were less likely to use a condom consistently. Our SEM results confirmed that condom use and psychological and personality factors were negatively associated, which is consistent with the findings of previous studies that used multiple logistic regression analyses (condom use vs loneliness, or condom use vs low self-esteem, or condom use vs depression) [36, 66, 67]. However, our finding that psychological and personality factors’ direct influence but nonexistence of an indirect influence is contrary to Nguyen’s findings [24]. The IMB model in Nguyen’s study was modified by an additional construct labelled ‘psychosocial factors’, which eventually showed an indirect influence but nonexistence of a direct influence. We believe this difference may be explained by several reasons. First, the additional construct did not consist of the same variables. The psychosocial factors in Nguyen’s study included some variables from our additional construct (e.g., depression) and was not limited to the psychological and personality factors in our study (e.g., low access to aids prevention, or alcohol use). Moreover, the two studies’ target populations were different, with one focused on male street laborers in Vietnam, and the other on unmarried female migrants in China. Despite these differences, both studies showed a significant effect of additional constructs on condom use, thus providing alternative variables for other researchers attempting to modify the IMB model in predicting condom use.

Examination of the mediation effect suggested the relation between motivation and CCU is fully mediated by behavior skill. Additionally, we found that in the modified IMB model, the indirect effect of motivation was larger than the direct effect of psychological and personality factors, thus suggesting prioritization of intervention targeted at motivation and behavioral skills. Although the effect of psychological and personality factors was small, it influenced CCU directly, which indicates another effective target for intervention. Our findings suggest that a shift from information-motivation-behavioral skills-focused interventions to a more comprehensive strategy that addresses psychological factors may be necessary.

Both the IMB model and the modified IMB model can be used to understand CCU in unmarried rural-to-urban female migrants in Shanghai. Overall, we tend toward adopting the modified IMB model because our purpose is not to find a hypothetical model that best matches the observed data, but to explore the rationality and appropriateness of building a model based on theory and the results of previous research.

The results of our study should be interpreted within the context of study limitations. First, the data from the two chosen factories may not be representative of unmarried rural-to-urban female migrants in other regions. Because migrants are generally mobile, obtaining random samples is difficult. Therefore, we used convenience sampling, which limited the representativeness of our sample. Second, data were gathered from a self-report questionnaire, so the reliability of the responses to sensitive questions may be questionable. There may exist some information bias. For example, “never” and “always” may be selected when measuring condom use even if they are not accurate. We minimized this problem by implementing various interventions and tools such as using anonymous questionnaires and providing private rooms for the survey. Third, due to the design’s cross-sectional nature, measures of information, motivation, behavioral skills, and the personality and psychological variables are assessed contemporaneously with consistent condom use, which made it difficult to decide the causality. This also limits the ability to control for habitual or past behavior - such as the amount of past sex or number of partners. Fourth, we noticed the relevance of power dynamics between men and women, and the subordinate position taken by female migrants incontraceptive use after the implementation of the project. It would be better to set up a variable that measures the subordinate position of female migrants in relationship. Fifth, the latent variable “psychological and personality factors” didn’t contain all the variables that may influence CCU. We preliminary include three variable because 1) migrants had been found to be more susceptible to them; 2) these three variables had been found correlated with CCU and 3) these three variables had also been found related to IMB model constructs. Further researches can be conducted to explore more variables related to psychological and personality factors. Sixth, combining three variables into one “psychological and personality factors” variable appeared to limit straightforward interpretation of the results because each variable may have different influences. However, separating these factors would complicate the model, which violates the rule of parsimony in structural equation modelling.

## Conclusions

In summary, our study is the first to demonstrate the utility of the modified IMB model for CCU among unmarried rural-to-urban female migrants and provide some new insights for future reproductive health promotion. We observed behavioral skill to be the main influencing factor of CCU. Additionally, motivation was also found to contribute to CCU indirectly by affecting behavioral skills. Moreover, psychological and personality factors were observed to have a negative direct influence on CCU. Further research promoting consistent condom use among unmarried rural-to-urban female migrants could develop preventive interventions not only on the basis of the IMB model, but also on the basis of psychological and personality factors.

## Abbreviations

AIDS: Acquired immunodeficiency syndrome
bootstrap BC: Bias-corrected bootstrap
BS: Behavior skills
CCU: Consistent condom use
CFI: Comparative fit index
HBM: Health Belief Model
HIV: Human immunodeficiency virus infection
IMB model: Information-motivation-behavioral skills model
MSM: Men who have sex with men
RMSEA: Root mean square error of approximation
SCT: Social Cognitive Theory
SEM: Structural equation modeling
STI: Sexually transmitted infections
TPB: Theory of Planned Behavior
TRA: Theory of Reasoned Action
